# Author Correction: Allelic variants of full-length VAR2CSA, the placental malaria vaccine candidate, differ in antigenicity and receptor binding affinity

**DOI:** 10.1038/s42003-022-03337-5

**Published:** 2022-04-26

**Authors:** Jonathan P. Renn, Justin Y. A. Doritchamou, Bergeline C. Nguemwo Tentokam, Robert D. Morrison, Matthew V. Cowles, Martin Burkhardt, Rui Ma, Almahamoudou Mahamar, Oumar Attaher, Bacary S. Diarra, Moussa Traore, Alassane Dicko, Niraj H. Tolia, Michal Fried, Patrick E. Duffy

**Affiliations:** 1 Laboratory of Malaria Immunology and Vaccinology, National Institute of Allergy and Infectious Diseases, National Institutes of Health, Bethesda, MD USA; 2 Malaria Research and Training Center, University of Sciences, Techniques, and Technologies of Bamako, Bamako, Mali

**Keywords:** Protein vaccines, Malaria

Correction to: *Communications Biology* 10.1038/s42003-021-02787-7, published online 19 November 2021.

An oversight was committed in neglecting to include several Malian co-authors who conducted the clinical study and provided samples for the study in the list of authors. This has now been corrected in the PDF and HTML versions of the Article.

Corrected list of authors and affiliations:

Jonathan P. Renn^1^, Justin Y.A. Doritchamou^1^, Bergeline C. Nguemwo Tentokam^1^, Robert D. Morrison^1^, Matthew V. Cowles^1^, Martin Burkhardt^1^, Rui Ma^1^, Almahamoudou Mahamar^2^, Oumar Attaher^2^, Bacary S. Diarra^2^, Moussa Traore^2^, Alassane Dicko^2^, Niraj H. Tolia^1^, Michal Fried^1^, Patrick E. Duffy^1^

1 Laboratory of Malaria Immunology and Vaccinology, National Institute of Allergy and Infectious Diseases, National Institutes of Health, Bethesda, MD, USA.

2 Malaria Research and Training Center, University of Sciences, Techniques, and Technologies of Bamako, Bamako, Mali.

