# Supplementary material for: Allelic variants of full-length VAR2CSA, the placental malaria vaccine candidate, differ in antigenicity and receptor binding affinity
Source: Commun Biol. 2021 Nov 19;4:1309. doi: 10.1038/s42003-021-02787-7 (PMC8604988; doi:10.1038/s42003-021-02787-7)

Supplementary Table S1: Yields of full-length VAR2CSA with different promoters and cell lines.
Protein yields for each allele are reported for different HEK293 suspension cell lines combined with different expression plasmids. This table reports the values in Figure 2C.

293F+pIRES		Expi293+pIRES		Expi293+pHLSEC	
Allele	Yield (mg/L)	Allele	Yield (mg/L)	Allele	Yield (mg/L)
FCR3	0.6	NF54	5	FCR3	17
FCR3	1.6	HB3	14	FCR3	19
NF54	0.7	7G8	4.5	NF54	13
HB3	1.5	M. Camp	1.6	NF54	16.6
7G8	2.5	M920	12	NF54	6.1
7G8	0.25	M200101	2.2	HB3	3.6
M. Camp	2.3			HB3	7.1
				7G8	10
				7G8	2.9
				M. Camp	7
				M. Camp	10.6

Supplementary Figure S1: Biacore data demonstrating full-length VAR2CSA binds to CSA (decorin).
Biacore traces of the response units at different protein concentrations that were used to calculate the steady state binding constant of VAR2CSA to decorin.

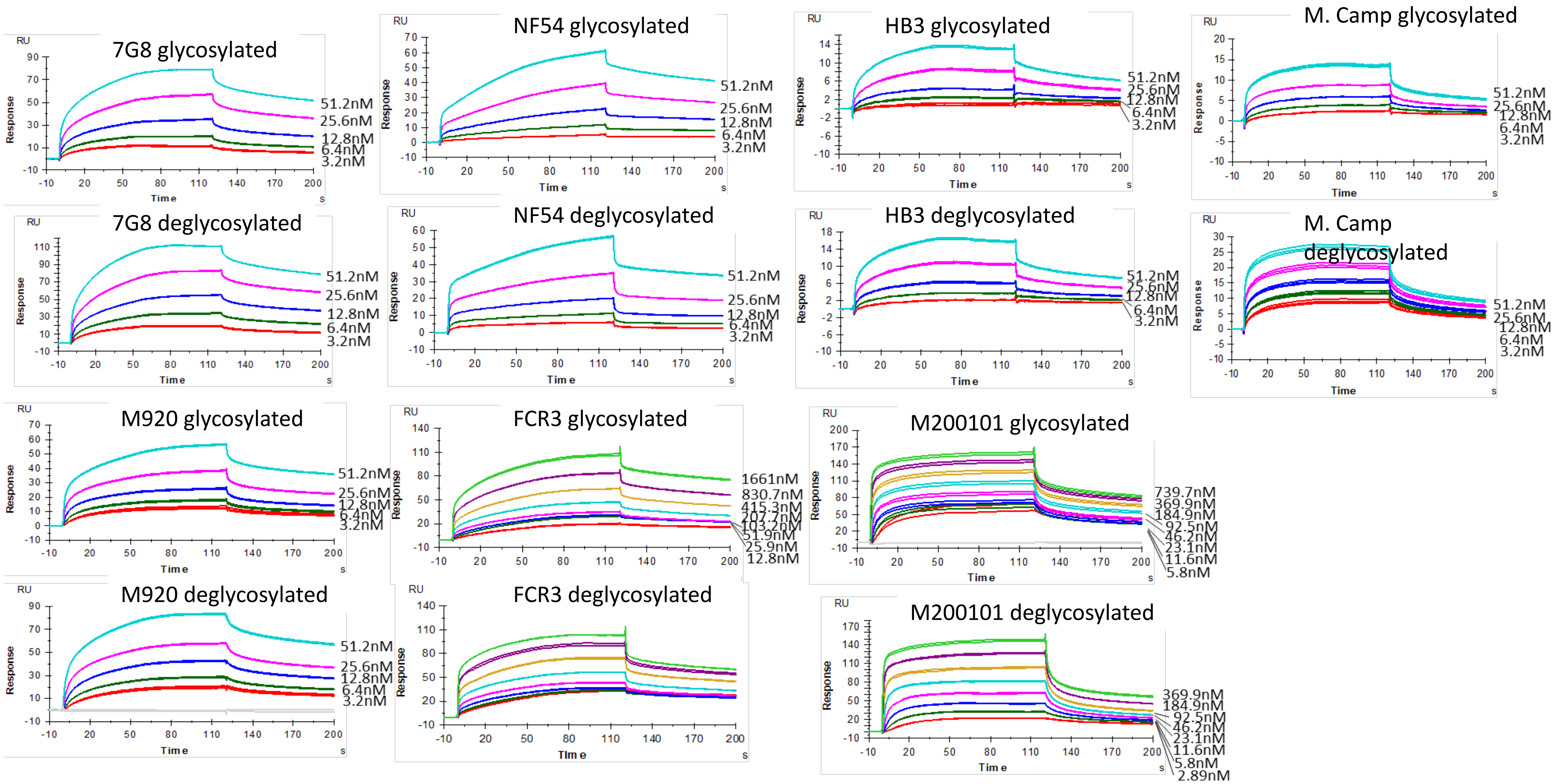

Supplementary Figure S2: Binding properties of recombinant full-length VAR2CSA proteins to CSA (decorin) as measured by ELISA.
ELISA plates were coated with decorin and serial diluted full-length VAR2CSA (FCR3=blue, NF54=red, HB3=purple, 7G8=yellow, M. Camp=green, M920-orange, M200101=black) was allowed to react on the plate then detected by an antibody that recognized the his-tag.

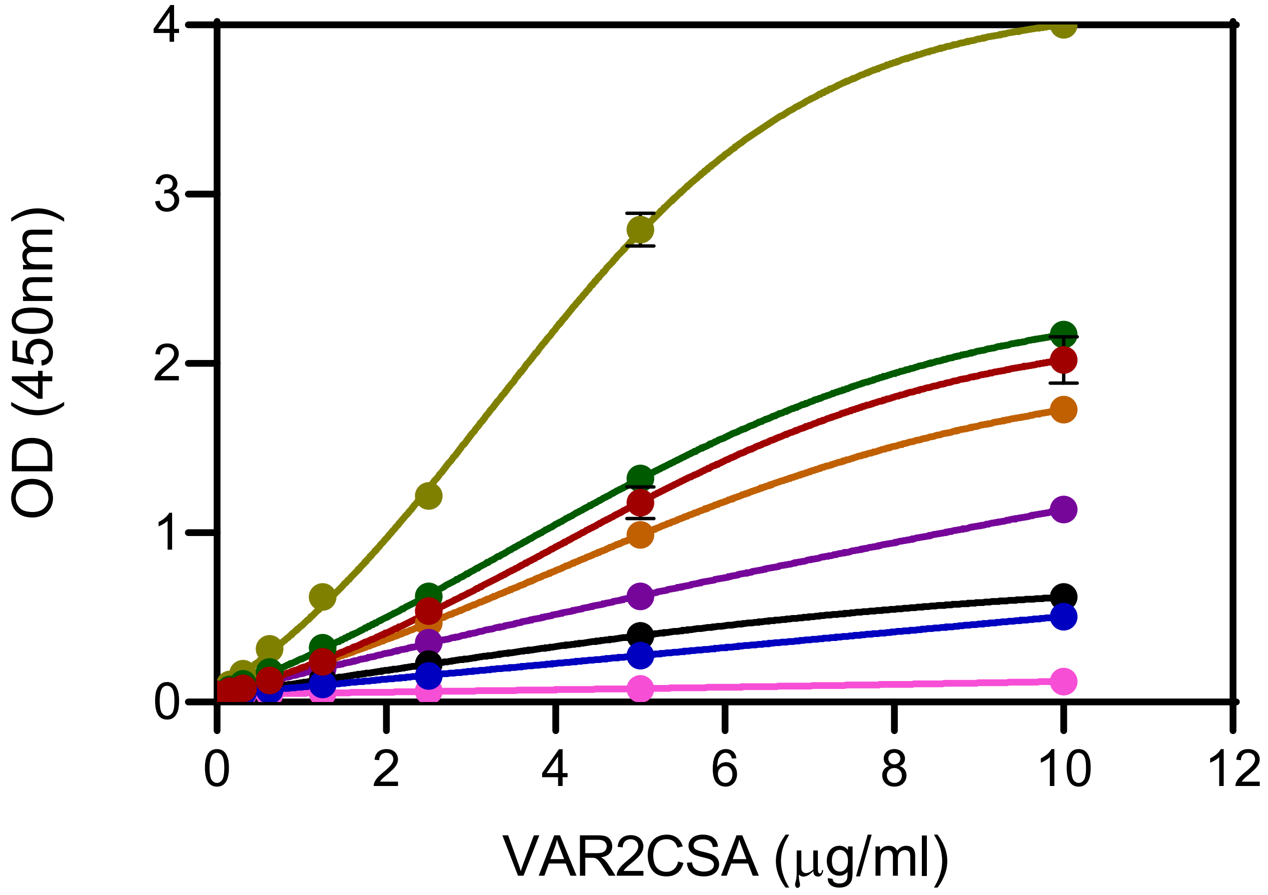

Supplementary Figure S3: Full-length VAR2CSA recombinants bind specifically to decorin. ELISA plates were coated with decorin, CSC, or BSA and 5 µg/mL of full length VAR2CSA (FCR3=blue, NF54=red, HB3=purple, 7G8=yellow, M. Camp=green, M920-orange, M200101=black) was bound to the different receptors. Panel A is the glycosylated VAR2CSA, and Panel B is the deglycosylated VAR2CSA. The amount of bound full-length VAR2CSA was detected by an HRP conjugated anti-his-tag. C) To test the density of chondroitin sulfate displayed by adsorbed decorin vs. CSC, ELISA plates were coated with either decorin or CSC and the amount of chondroitin sulfate was measured by using 2 µg/mL of anti-chondroitin sulfate antibody that recognized both CSA and CSC.

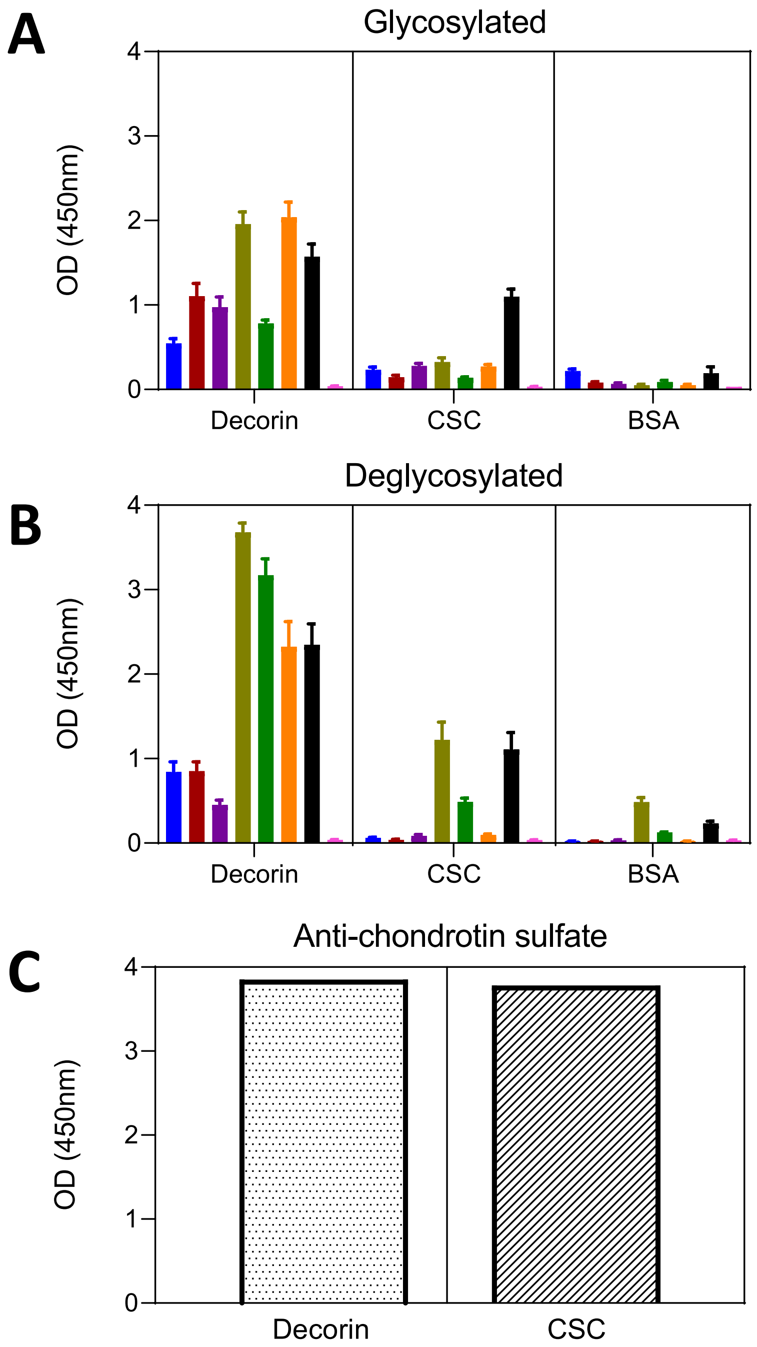

Supplementary Figure S4: Full-length glycosylated VAR2CSA recombinants are recognized by naturally acquired antibodies in gravidity-dependent manner. Box and Whisker representation of ELISA reactivity to glycosylated VAR2CSA from 150 samples stratified by gravidity (n=50 multigravida, n=50 secundigravida and n=50 primigravida) from malaria-exposed women and 50 US naïve samples. The black line represents the median of reactivity. Statistically significant differences between the parties are indicated with p values <0.05=*, <0.01=**, <0.001=***, and <0.0001=**** based on Mann-Whitney test.

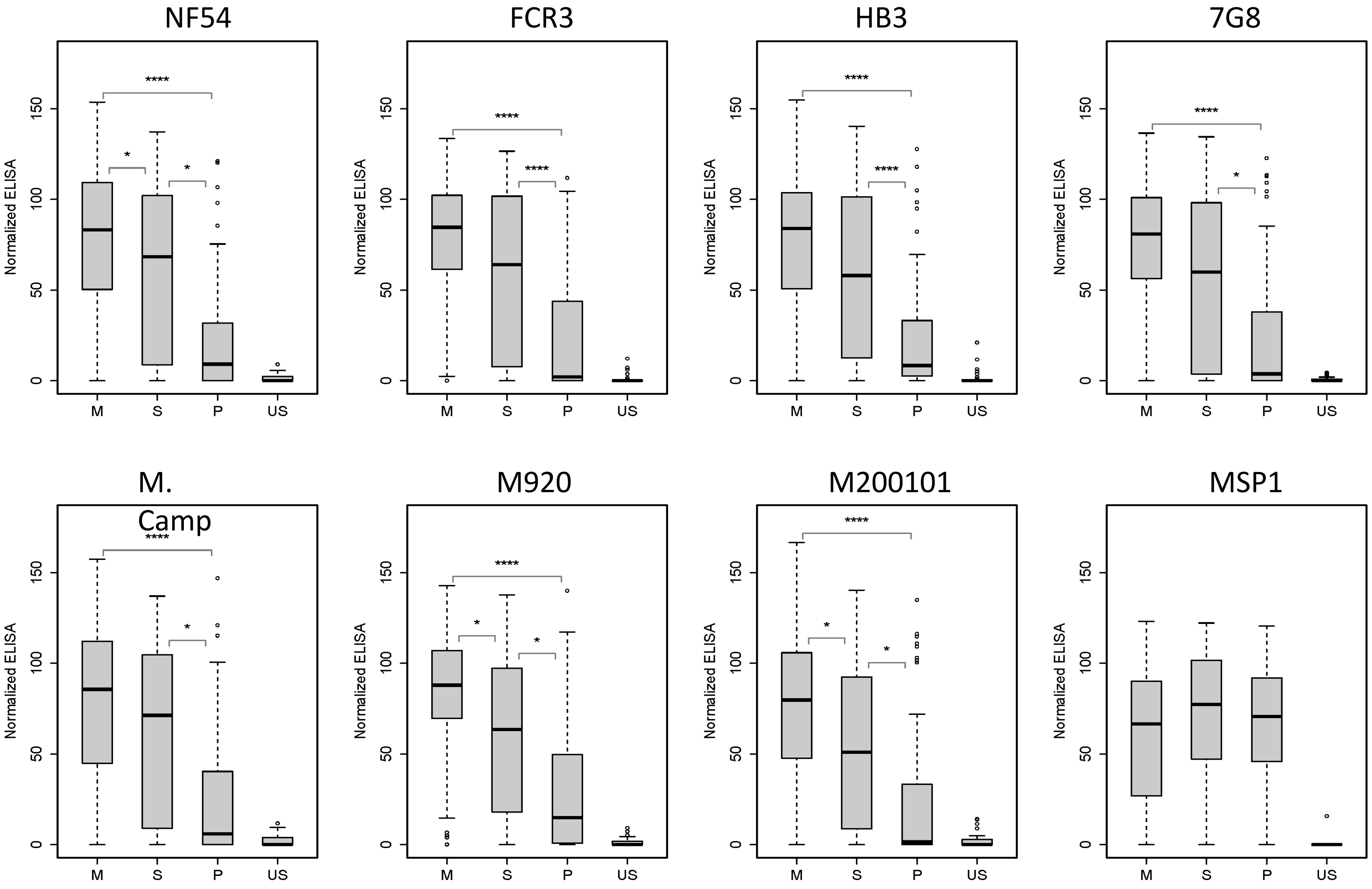

Supplementary Figure S5: Recognition of glycosylated full-length VAR2CSA recombinants by naturally acquired antibodies increases with trimester. Box and Whisker representation of ELISA reactivity to glycosylated VAR2CSA from 148 samples stratified by trimester (n=11 first, n=46 second, n=20 third, n=71 delivery) from malaria-exposed women and 50 US naïve samples. The black line represents the median of reactivity. Statistically significant differences between the parties are indicated with p values <0.05=*, <0.01=**, <0.001=***, and <0.0001=**** based on Mann-Whitney test.

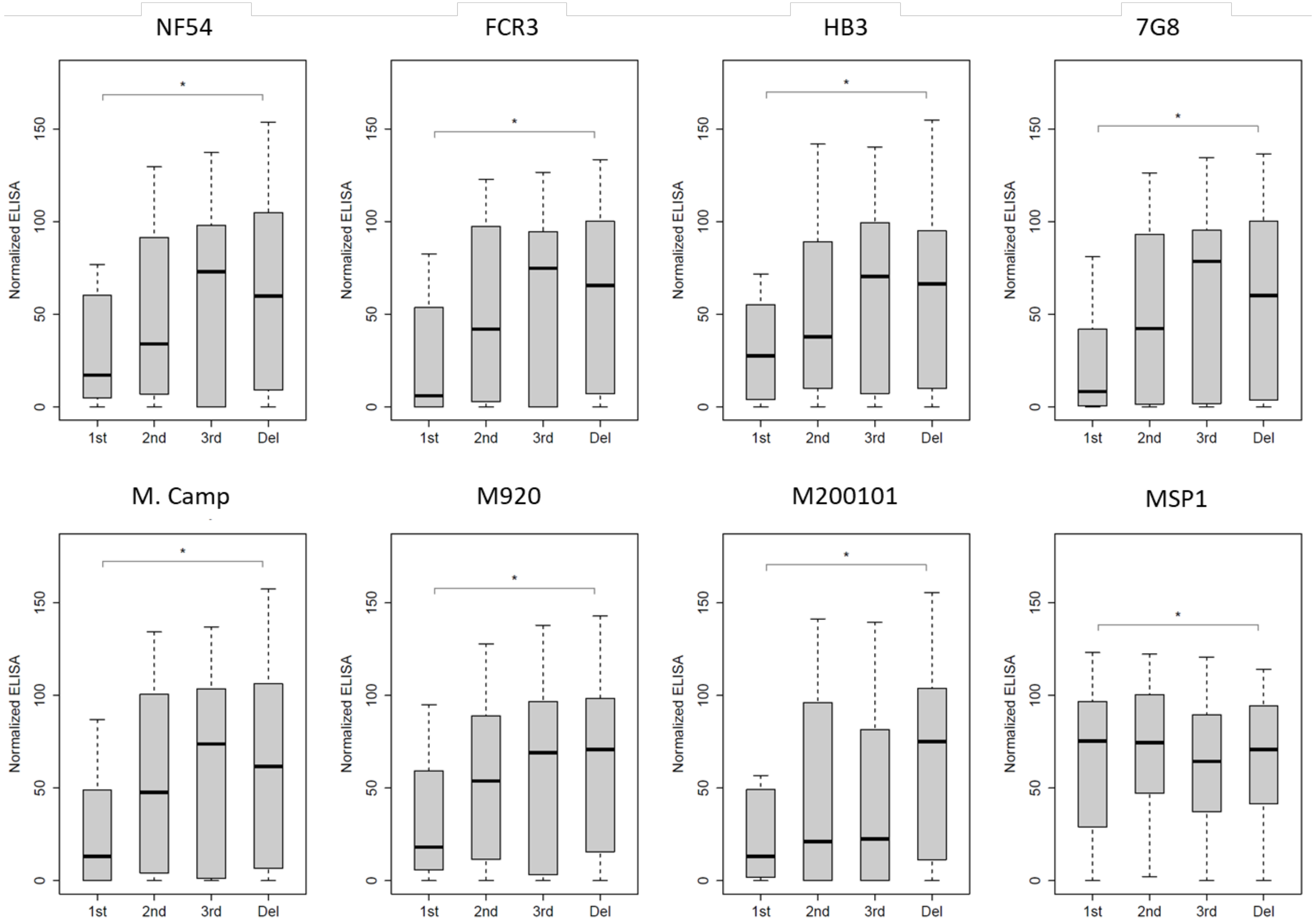

Supplementary Figure S6: Deglycosylated full-length VAR2CSA analyzed by size-exclusion chromatography and SDS-PAGE.
Panel A) Size exclusion analysis of all 7 full-length VAR2CSA proteins ran on a Superose 6 column. Each trace is in a different color (FCR3=blue, NF54=red, HB3=purple, 7G8=yellow, M. Camp=green, M920-orange, M200101=black). All proteins eluted around 15 mL (void volume of the column is 8 mL) compared to the glycosylated variants that eluted around 14 mL (Figure 2B). Panel B) Deglycosylated VAR2CSA recombinants were analyzed in SDS-PAGE gels. Ten µg of purified full-length VAR2CSA FCR3 (lane 1 and 9), NF54 (lane 2 and 10), 7G8 (lane 3 and 11), HB3 (lane 4 and 12) M. Camp (lane 5 and 13), M920 (lane 6 and 14), and 2 µg of M200101 (lane 7 and 15) were loaded on a 3–8% tris-acetate gel (with or without 100 mM DTT) and Coomassie stained. Lane 8 is the molecular weight marker (kDa).

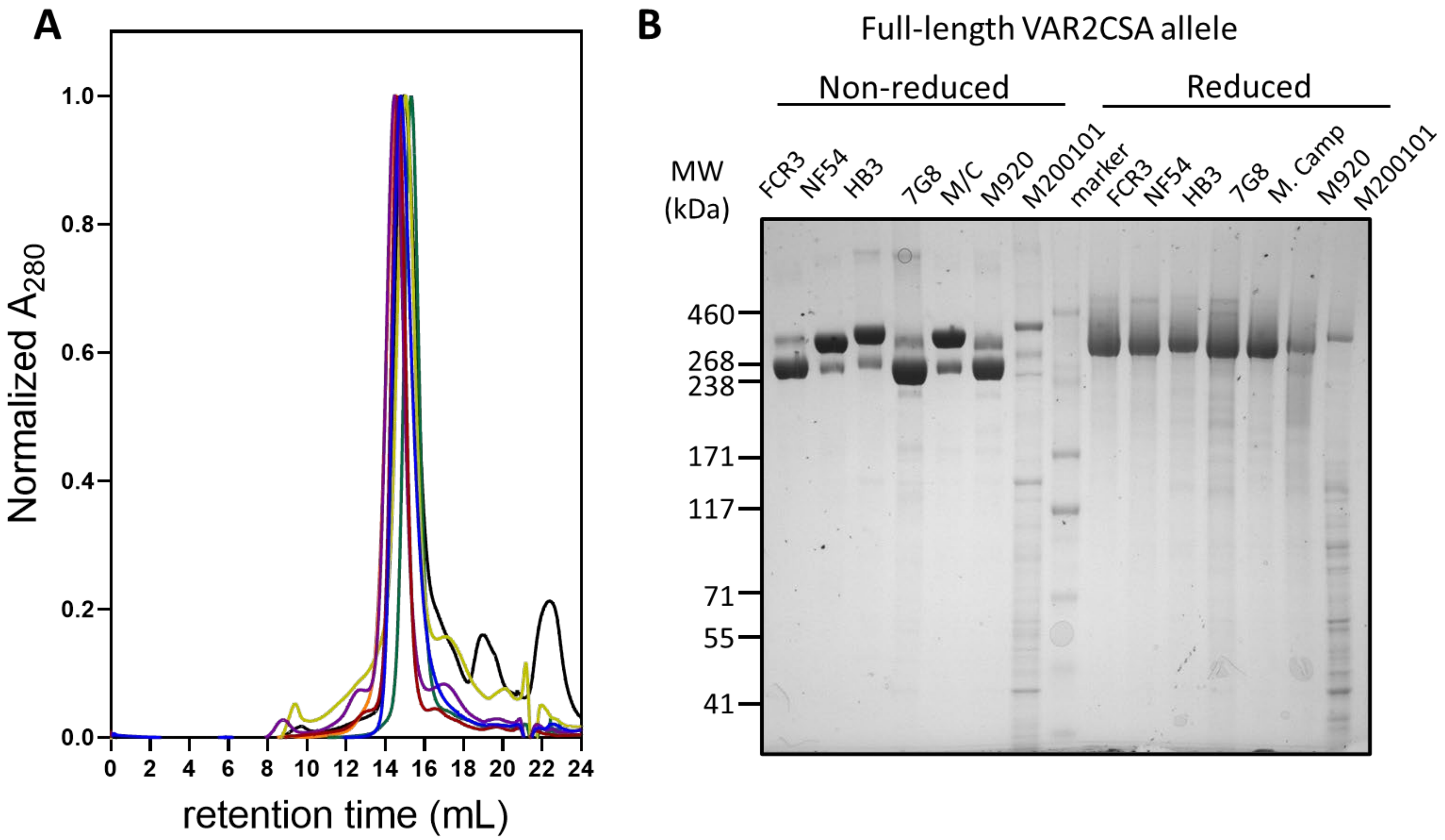

Supplementary Figure S7: Full-length deglycosylated VAR2CSA recombinants are recognized by naturally acquired antibodies in gravidity-dependent manner. Box and Whisker representation of ELISA reactivity to deglycosylated VAR2CSA from 150 samples stratified by gravidity (n=50 multigravida, n=50 secundigravida and n=50 primigravida) from malaria-exposed women and 50 US naïve samples. The black line represents the median of reactivity. Statistically significant differences between the parties are indicated with p values <0.05=*, <0.01=**, <0.001=***, and <0.0001=**** based off Mann-Whitney test.

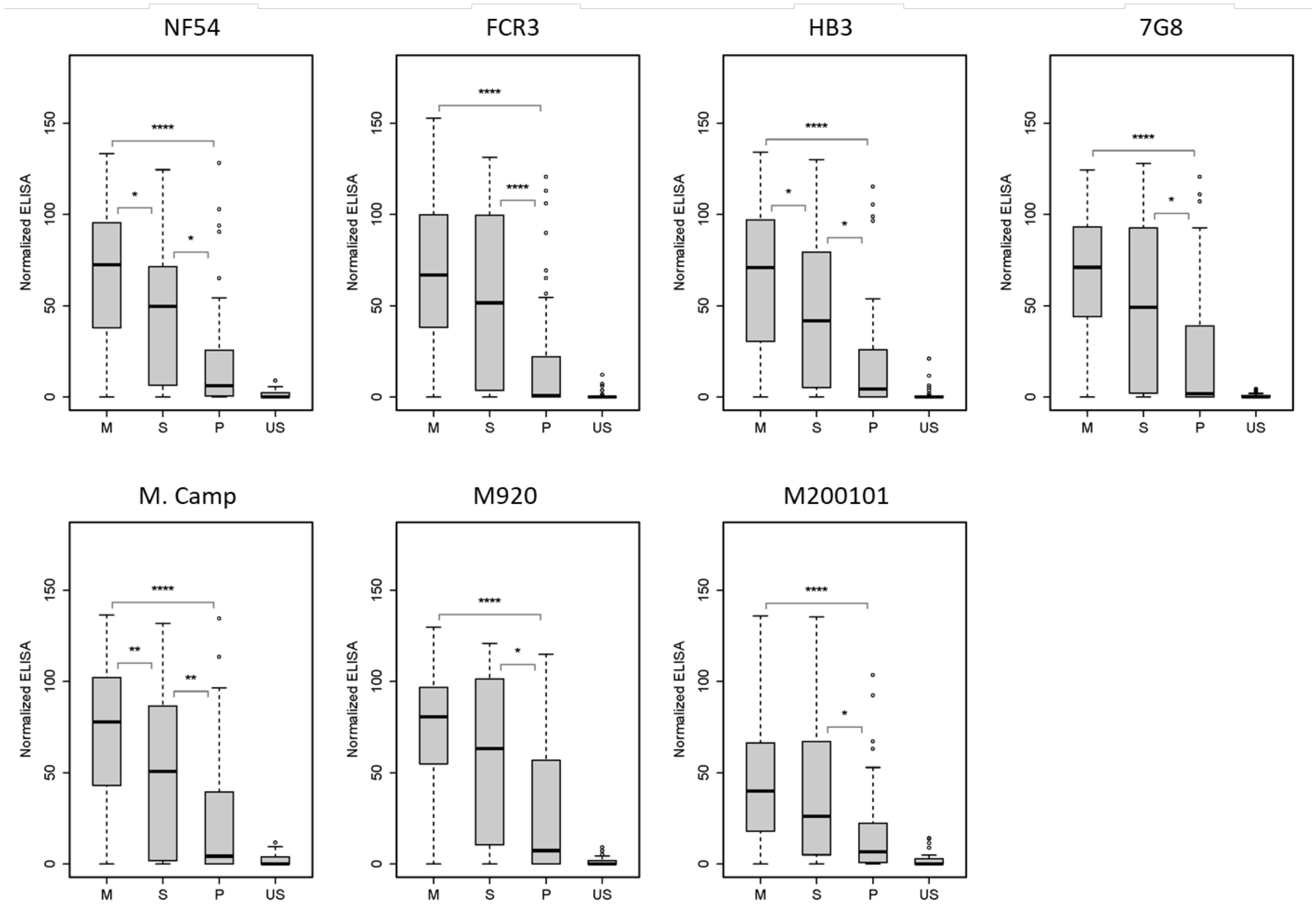

Supplementary Figure S8: Recognition of deglycosylated full-length VAR2CSA recombinants by naturally acquired antibodies increases with trimester. Box and Whisker representation of ELISA reactivity to deglycosylated VAR2CSA from 148 samples stratified by trimester (n=11 first, n=46 second, n=20 third, n=71 delivery) from malaria-exposed women and 50 US naïve samples. The black line represents the median of reactivity. Statistically significant differences between the parties are indicated with p values <0.05=*, <0.01=**, <0.001=***, and <0.0001=**** based off Mann-Whitney test.

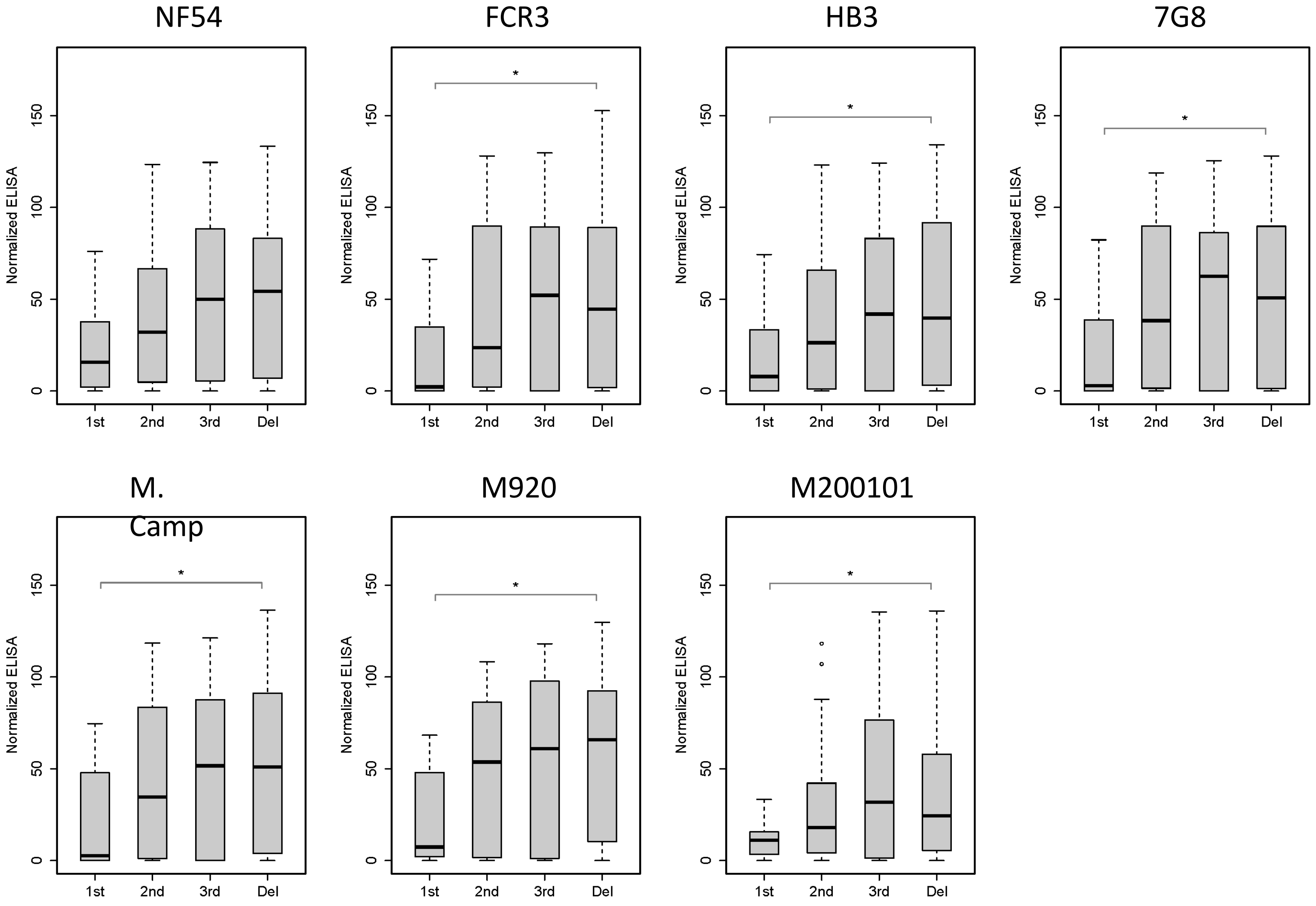

Supplementary Figure S9: Recognition of full-length VAR2CSA by naturally acquired antibodies varies by allele.

The ELISA reactivity of all 150 samples compared by allele (FCR3=blue, NF54=red, HB3=purple, 7G8=yellow, M. Camp=green, M920-orange, M200101=black). The solid line in the box and whisker plot is the media of the reactivity data. While a violin plot superimposed on the box and whisker plot shows the distribution of reactivity in each allele. The Kruskal-Wallis test was used to compare if allele reactivity differed significantly in each of the conditions (gravidity or glycosylation). Only the deglycosylated and multigravid group was significant by this method (p-value=0.006).

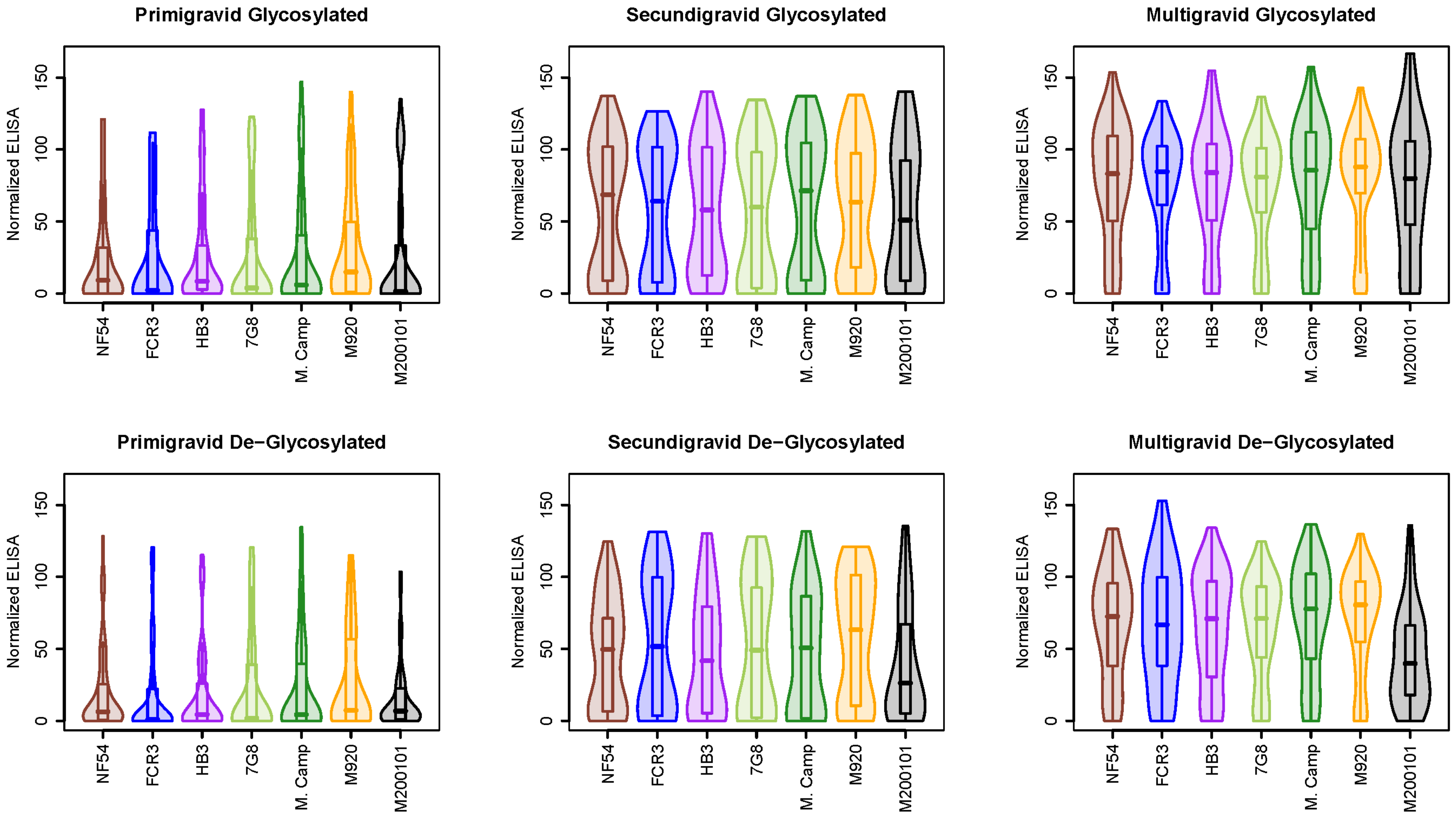

Supplementary Figure S10: P-values from comparisons of reactivity of naturally acquired antibodies to different alleles of full-length VAR2CSA. Heat maps displaying the p-values from the Wilcoxon signed-rank test comparing ELISA reactivity to naturally-acquired antibodies to different pairs of VAR2CSA. The p-values of the allele comparisons were adjusted for multiple comparisons using the Benjamini and Hochberg method. Significant comparisons are shaded in red with the p-value displayed. The increasing the shade of red indicates the more significant the p-value. The data has been stratified by gravidity and glycosylation state.

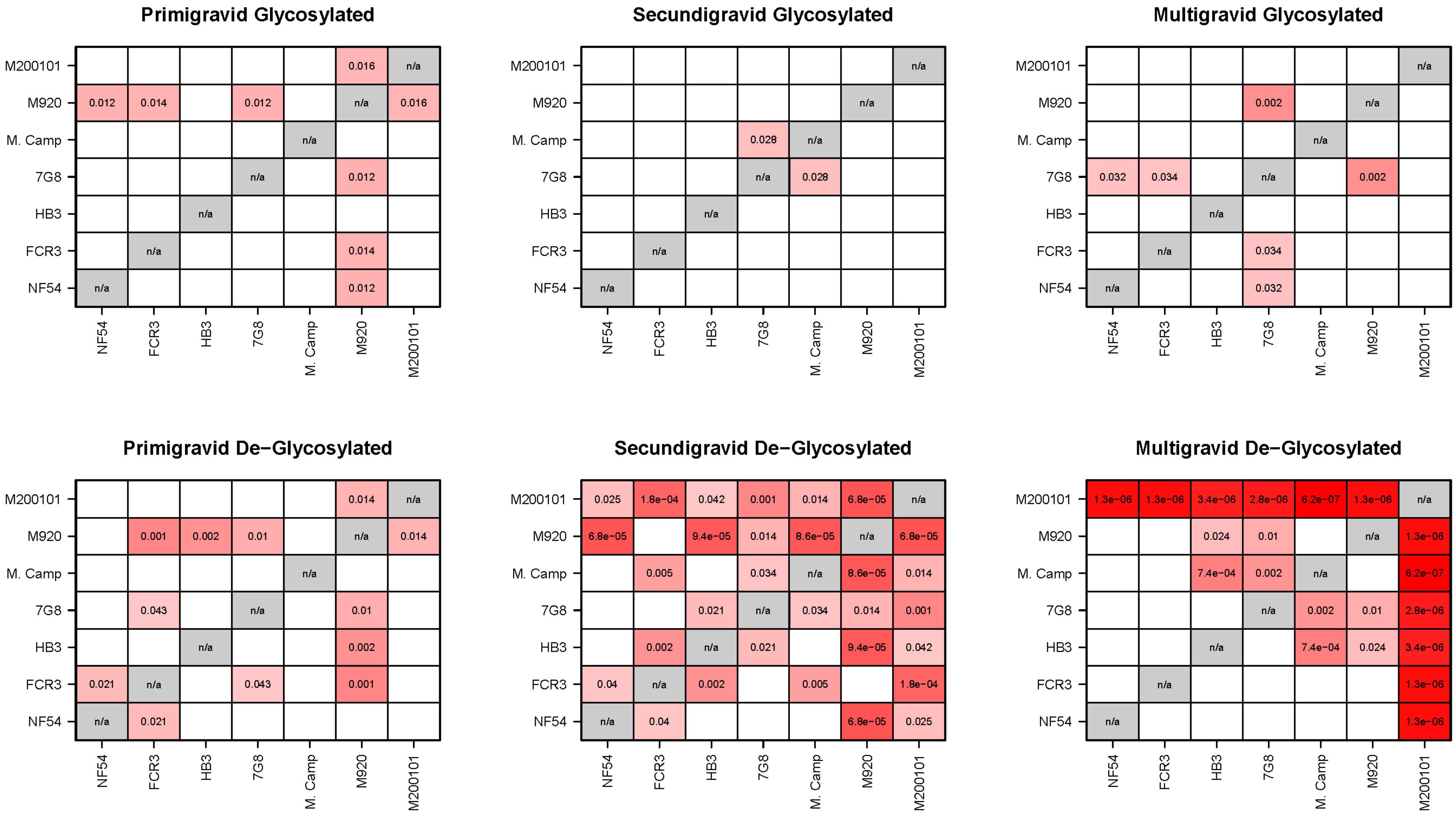

Supplement: Supplementary file 2 — Supplementary Material [file 42003_2021_2787_MOESM2_ESM.pdf]
