# Supplementary material for: Allelic variants of full-length VAR2CSA, the placental malaria vaccine candidate, differ in antigenicity and receptor binding affinity
Source: Commun Biol. 2021 Nov 19;4:1309. doi: 10.1038/s42003-021-02787-7 (PMC8604988; doi:10.1038/s42003-021-02787-7)
Supplement: Supplementary file 3 — Description of Additional Supplementary Files [file 42003_2021_2787_MOESM3_ESM.pdf]

Description of Additional Supplementary Files

File name: Supplementary Data 1

Description: Figure source data

File name: Supplementary Data 2

Description: Biacore source data
