# Supplementary material for: Allelic variants of full-length VAR2CSA, the placental malaria vaccine candidate, differ in antigenicity and receptor binding affinity
Source: Commun Biol. 2021 Nov 19;4:1309. doi: 10.1038/s42003-021-02787-7 (PMC8604988; doi:10.1038/s42003-021-02787-7)
Supplement: Supplementary file 6 — Reporting Summary [file 42003_2021_2787_MOESM6_ESM.pdf]

nature portfolio

Corresponding author(s): *Double-blind peer review submissions: write DBPR and your manuscript number here instead of author names.*

Last updated by author(s): *YYYY-MM-DD*

Reporting Summary

Nature Portfolio wishes to improve the reproducibility of the work that we publish. This form provides structure for consistency and transparency in reporting. For further information on Nature Portfolio policies, see our Editorial Policies and the Editorial Policy Checklist.

Statistics

For all statistical analyses, confirm that the following items are present in the figure legend, table legend, main text, or Methods section.

n/a	Confirmed	
☐	☒	The exact sample size (*n*) for each experimental group/condition, given as a discrete number and unit of measurement
☐	☒	A statement on whether measurements were taken from distinct samples or whether the same sample was measured repeatedly
☐	☒	The statistical test(s) used AND whether they are one- or two-sided<br>*Only common tests should be described solely by name; describe more complex techniques in the Methods section.*
☒	☐	A description of all covariates tested
☒	☐	A description of any assumptions or corrections, such as tests of normality and adjustment for multiple comparisons
☒	☐	A full description of the statistical parameters including central tendency (e.g. means) or other basic estimates (e.g. regression coefficient) AND variation (e.g. standard deviation) or associated estimates of uncertainty (e.g. confidence intervals)
☒	☐	For null hypothesis testing, the test statistic (e.g. *F*, *t*, *r*) with confidence intervals, effect sizes, degrees of freedom and *P* value noted<br>*Give P values as exact values whenever suitable.*
☒	☐	For Bayesian analysis, information on the choice of priors and Markov chain Monte Carlo settings
☒	☐	For hierarchical and complex designs, identification of the appropriate level for tests and full reporting of outcomes
☒	☐	Estimates of effect sizes (e.g. Cohen's *d*, Pearson's *r*), indicating how they were calculated

Our web collection on statistics for biologists contains articles on many of the points above.

Software and code

Policy information about availability of computer code

Data collection	Biacore T200 Evaluation Software<br>Jasco Spectra Manager Suite Spectroscopy Software for CD spectroscpy data collection
Data analysis	GraphPad Prism Version 8 was used for graphing and data analysis<br>R version 3.6.3 was used for statistics and analysis and visualization of the ELISA

For manuscripts utilizing custom algorithms or software that are central to the research but not yet described in published literature, software must be made available to editors and reviewers. We strongly encourage code deposition in a community repository (e.g. GitHub). See the Nature Portfolio guidelines for submitting code & software for further information.

Data

Policy information about availability of data

All manuscripts must include a data availability statement. This statement should provide the following information, where applicable:

- Accession codes, unique identifiers, or web links for publicly available datasets
- A description of any restrictions on data availability
- For clinical datasets or third party data, please ensure that the statement adheres to our policy

The raw FASTQ sequences of M920 and Malayan Camp isolates presented in this article have been submitted to the Gene Expression Omnibus (www.ncbi.nlm.nih.gov/geo). The source data plotted in figures are provided in Supplementary Data. All other data relevant to this study are available from the corresponding author on reasonable request.

March 2021

Field-specific reporting

Please select the one below that is the best fit for your research. If you are not sure, read the appropriate sections before making your selection.

☒ Life sciences ☐ Behavioural & social sciences ☐ Ecological, evolutionary & environmental sciences

For a reference copy of the document with all sections, see nature.com/documents/nr-reporting-summary-flat.pdf

Life sciences study design

All studies must disclose on these points even when the disclosure is negative.

Sample size	No sample-size calculation was performed for this work.
Data exclusions	No data was excluded
Replication	Replication experiments were successful
Randomization	Samples for ELISA were selected 2 longitudinal cohorts that were separated by gravidity. Then 50 random samples were selected from each gravidity group were selected for the study.
Blinding	The ELISA measurements were performed blinded.

Reporting for specific materials, systems and methods

We require information from authors about some types of materials, experimental systems and methods used in many studies. Here, indicate whether each material, system or method listed is relevant to your study. If you are not sure if a list item applies to your research, read the appropriate section before selecting a response.

Materials & experimental systems

n/a	Involved in the study	
☐	☒	Antibodies
☐	☒	Eukaryotic cell lines
☒	☐	Palaeontology and archaeology
☒	☐	Animals and other organisms
☐	☒	Human research participants
☒	☐	Clinical data
☒	☐	Dual use research of concern

Methods

n/a	Involved in the study	
☒	☐	ChIP-seq
☒	☐	Flow cytometry
☒	☐	MRI-based neuroimaging

Antibodies

Antibodies used	Anti-Chondroitin Sulfate antibody [CS-56] (ab11570) from Abcam. Goat Anti-Mouse IgM mu chain (HRP) (ab97230) from Abcam. 6x-His Tag Monoclonal Antibody (HIS.H8), HRP, 6-His Tag Antibody, A190-114P from Bethyl Laboratories , Goat anti-Human IgG Fc - Affinity Pure, HRP Conjugate from ImmunoReagents INC.
Validation	Goat Anti-Mouse IgM mu chain (HRP) (ab97230) from Abcam, from the manufacturers website: By immunoelectrophoresis and ELISA this antibody reacts specifically with Mouse IgM. Cross reactivity with other immunoglobulins and light chains is less than 0.1%.<br>6x-His Tag Monoclonal Antibody (HIS.H8), HRP was validated in a previous publication, see Doritchamou et. al. Commun Biol. 2019 Dec 6;2:457<br>6-His Tag Antibody, A190-114P from Bethyl Laboratories was validated in this manuscript in ELISA against his tagged proteins<br>Anti-Chondroitin Sulfate antibody [CS-56] (ab11570) from Abcam was validated in this manuscript in ELSIA against CSC and CSA<br>Goat anti-Human IgG Fc - Affinity Pure, HRP Conjugate from ImmunoReagents INC. was validated by the manufacture. According to the manufactures website: Based on IEP, no reactivity is observed to: non-immunoglobulin human serum immunoglobulins light chains on all human immunoglobulins and Based on IEP, this antibody reacts with: heavy (γ) chains on human IgG. In the COA the antibody is tested in ELISA: Dilution giving A450 reading of ≥ 1.0 when tested against Human IgG (H&L) coated at 2 µg/ml.

Eukaryotic cell lines

Policy information about cell lines

Cell line source(s)	HEK293F and Expi293
Authentication	Cells are commercially available and authenticated according to the manufacturer. (see https://www.thermofisher.com/

order/catalog/product/A14527?SID=srch-srp-A14527 and https://www.thermofisher.com/order/catalog/product/R79007?SID=srch-srp-R79007#/R79007?SID=srch-srp-R79007)

Mycoplasma contamination	Cells lines were not tested for mycoplasma contamination during the expression of the recombinant proteins used in this work. The cell stocks are purchased from ThermoFisher as mycoplasma free and our stocks are made from early passage from purchased stock. Mycoplasma contamination test performed for cultures in the lab were negatives.
Commonly misidentified lines (See ICLAC register)	No commonly misidentified cell lines were used in this study

Human research participants

Policy information about studies involving human research participants

Population characteristics	Briefly, pregnant women were enrolled between November 2010 and October 2013 into a longitudinal cohort study of mother-infant pairs conducted in Ouélessébougou, Mali. The study site is located 80 km south of Bamako, an area of intense seasonal malaria transmission during the rainy season from July to December.
Recruitment	Pregnant women aged 15–45 years without clinical evidence of chronic or debilitating illness were asked to participate in the study and gave signed informed consent after receiving a study explanation form and oral explanation from a study clinician in their native language.
Ethics oversight	The protocol and study procedures were approved by the institutional review board of the National Institute of Allergy and Infectious Diseases at the US National Institutes of Health, and the Ethics Committee of the Faculty of Medicine, Pharmacy and Dentistry at the University of Bamako, Mali.

Note that full information on the approval of the study protocol must also be provided in the manuscript.
